# How Art Museums Can Foster the Flourishing of Future Physicians

**DOI:** 10.5334/pme.1905

**Published:** 2025-08-07

**Authors:** Winnie L. Liu, Margaret S. Chisolm

**Affiliations:** 1 Johns Hopkins University School of Medicine, Baltimore, Maryland, US

## Abstract

Medical schools are increasingly incorporating the arts and humanities into their curricula, often emphasizing their benefits in enhancing students’ clinical skills. However, the potential for these curricula to foster flourishing is equally, if not more, crucial. Although limited in scope, current literature suggests that art museum-based medical education, particularly as Visual Thinking Strategies, may support flourishing among medical learners—although more research is needed to assess the impact of these methods and their mechanisms on learners. In this article, the authors explore how integrating art museum-based programs into medical school curricula can enhance all five domains of the VanderWeele model of human flourishing: physical and mental health, happiness and life satisfaction, meaning and purpose, character and virtue, and close social relationships.

## Introduction

Today’s medical students must master an ever-expanding body of knowledge while navigating earlier clinical clerkships, residency applications, and other student pressures (e.g., rising tuition, family obligations) [1,2,3]. Moreover, some educators have argued that the current curriculum excels at helping students “know” medicine, but lacks in guiding students’ journeys towards “becoming a good doctor” and discovering their values [4]. In the face of these challenges, medical educators have begun to call for, and implement, approaches and models that help students not just survive their training, but genuinely flourish as resilient, empathic, and healthy future physicians [4,5,6].

Flourishing has been defined as “the relative attainment of a state in which all aspects of a person’s life are good, including the contexts in which that person lives” [7,8]. Many models currently exist to conceptualize flourishing, such as the PERMA model proposed by Martin Seligman, and to assess how the arts contribute to flourishing, such as the Tay, Pawelski, and Keith conceptual model [9,10]. In this article, we have chosen to focus on the model developed by VanderWeele [14] for several reasons: 1) VanderWeele’s flourishing model identifies several interrelated domains of flourishing, each of which is a universally desired good that is an end in itself: mental and physical health, happiness and life satisfaction, meaning and purpose, character and virtue, and close social relationships; 2) VanderWeele’s model encompasses those aspects of flourishing around which there is general consensus and focuses on domains that educators can be prepared to support—or that can be adapted to become feasible with appropriate training and resources; [11] 3) VanderWeele’s model is currently being used in the Global Flourishing Study, which is measuring flourishing longitudinally in a representative sample of half of the world’s population [12,13]. Of note, although each of the five principal domains of flourishing are considered ends in themselves (i.e., of intrinsic value), they are not only ends. The domains are also means to ends (i.e., of instrumental value). For example, happiness is a desired end in itself, but happiness also contributes on average to better relationships and health (among other instrumental purposes) and so is a means to an end as well [14]. Likewise, close relationships are valued in their own right, but are also of instrumental value to improving health, happiness, and meaning (when viewed as means/instrumental value, relationships are often referred to as “social support” or “social capital” and when of ends/intrinsic value as “social connection”) [14].

Art has been recognized as a powerful medium—capable of evoking emotion, inspiring reflection, increasing association with well-being, and fostering genuine connections [15,16]. Art museum-based medical education offers an innovative way to nurture students’ core dimensions of flourishing. Many museum-based pedagogies currently exist, such as the Personal Responses Tour, mask-making, and creation of group poems [17,18,19,20,21]. One of the methods with the greatest evidence is Visual Thinking Strategies (VTS), which provides a structured framework for facilitating open-ended discussions prompted by a work of art, ensuring methodological consistency while allowing room for creativity [22,23,24]. Whether conducted in the classroom, online, or in a museum, VTS and other museum-based pedagogical methods encourage students to use works of art to explore the big questions—what it means to be human, to be a physician, and to lead a good life (for themselves and their patients) [23,25]. In this paper, we explore how art museum-based learning activities can support medical learners’ flourishing across all five domains of VanderWeele’s model. We argue that incorporating art museum-based learning into medical education can help students flourish into physicians who are not only clinically excellent, but also deeply connected to their own humanity and that of their patients.

## Limitations of Traditional Medical Education

Traditional medical education excels at helping students refine their biomedical expertise, analyze data, perform basic procedures, and follow diagnostic algorithms or protocols. Today, an increasing number of medical curricula are focused on supporting student competencies that extend beyond the acquisition of knowledge and technical skills [26,27,28,29]. Medical learners may diagnose advanced conditions yet struggle to detect subtle emotional cues in a patient’s demeanor. They may falter in navigating ambiguous situations requiring deviation from algorithms. They may lose sight of their original motivations to become a physician and find themselves failing to connect meaningfully with patients. The demanding nature of the curriculum may not allow students enough time to reflect on emotionally powerful experiences such as processing the death of their first patient. Students may not have the time or support to encourage them to consider alternative perspectives or potential biases. They may find themselves unable to examine the ethical dimensions of care. They may be unable to prioritize their own self-care. While technical competence is a nonnegotiable aspect of clinical care, the complementary humanistic understanding can help students find the time, space, and support they need to reflect, empathize, and flourish in both their personal and professional lives [30]. In recent years, medical education has put more emphasis on humanistic development and has advocated for greater integration of arts and humanities into the curriculum to support these goals [6,31].

## Promoting Mental and Physical Health

Art museum-based education is defined as an “approach that can occur in museums, other in-person settings, and/or online, which is informed by museum-based education adapted for health professional learners and involves exploring and/or creating visual and other forms of art, as well as individual and group reflection on these activities” [32]. Art museum-based education may foster emotional regulation and mindfulness among medical learners, as well as offer them an emotional reprieve from the demands of training. This may be because viewing artwork, in general, can be beneficial to reducing stress as suggested by a recent scoping review [33]. That review examined the effects of viewing artwork on stress outcomes in museum visitors and found a reduction in self-reported stress levels in 13 out of 14 of the included studies and reduced cortisol levels in three of the included studies [33]. Additionally, a reduction in systolic blood pressure was found in the four studies that measured this parameter, suggesting that engaging with art may have physiological influences as well. Although these studies have limitations and did not all present artwork specifically in a museum, they suggest that viewing artwork and utilizing museum-based approaches may be beneficial to well-being.

Beyond stress markers, key elements of art museums make them welcoming spaces conducive to increasing well-being factors and flourishing [16]. Many museums are designed to foster comprehensibility and comfort through clearly labeled exhibits, guided prompts, and/or educators or interactive exhibits to facilitate discussion and, ultimately, visitor well-being [34]. Further, art museums can encourage engagement with (and tolerance of) unfamiliar material while also encouraging reflections that connect personal insights to broader cultural or historical contexts [35,36]. Museums can also offer freedom from the pressures to be correct, as unlike clinical or academic environments, no expectation of reaching a predetermined “right answer” to the artwork exists [35]. And unlike self-reflection only, which can carry internalized pressure to be self-critical, museums provide a safe space that invites exploration alongside peers’ diverse perspectives in a setting of respect and confidentiality [37,38].

Overall, museum-based learning activities may also offer an added advantage over one-on-one feedback, which students can experience as evaluative and result in a reduced sense of psychological safety, especially if the supervising facilitator is in a higher perceived position in hierarchy [39]. Art museum-based pedagogy may also complement narrative medicine sessions, which often rely heavily on verbal or written expression and may lack the multisensory, spatial, and interpretive freedom of a museum experience that anchor the learning into memory [38]. A prior study has found this approach to be beneficial to improving not only students’ capacity for written reflection, but also their capacity for wonder [40]. Therefore, museum-based approaches can be synergistic to other powerful reflection-based strategies, adding further support for their increased integration into medical education [6,31].

The Personal Responses Tour, for example, is a museum-based pedagogical approach in which participants are invited to select artworks in response to personally meaningful prompts, encouraging emotional expression and empathetic dialogue [19]. Unlike the high-stakes academic and clinical environments of medical education and training, which focus on rigid evaluations and rapid “right vs. wrong” decisions, museum-based activities offer the kind of psychological safety that allows students to process emotions, discuss ‘taboo” topics, or voice imperfect thoughts without judgment [19]. This freedom may further promote emotional regulation, as students are able to identify similar emotions in artwork, confront them, and understand those feelings in a non-isolated, supportive environment. Given that the nature of care delivery can still influence medical learners, and even established physicians, to suppress their emotions of grief or perpetuate self-blame so that all efforts are focused on delivering optimal care to every patient [41], activities such as interactive exhibits or group art-discussions grounded in Visual Thinking Strategies (VTS) provide a much-needed pause for contemplation and decompression [24,40,42].

Engaging with art may also promote emotional intelligence and self-awareness [43,44]. In the context of art museum-based medical education, this may occur by encouraging students to explore universal themes depicted in artwork, and possibly relate them to their own experiences. While not an exhaustive list, the following initiatives illustrate how art museum-based courses have been incorporated into medical curricula to deepen clinical training through reflective and emotionally meaningful engagement. Programs like the Baltimore Museum of Art’s reflective workshops in collaboration with Johns Hopkins School of Medicine incorporate pieces like Vincent van Gogh’s *Ravine*, inviting students to reflect on themes of hope amidst an amorphous landscape and connect them to their personal journeys through the COVID-19 pandemic [45]. Similarly, the Sticky Questions workshop, developed at the University of Calgary, features a provocative, discomforting painting designed to help healthcare students discuss and process topics such as vulnerability, power dynamics, and patient dignity [46].

## Happiness and Life Satisfaction

Research has found that engaging with art, including observing artwork and visiting museums, is associated with improved life satisfaction, including when controlling for age, gender, and monthly income [47]. Similarly, curiosity and wonder have been associated with greater happiness and intellectual satisfaction. While curiosity increases with uncertainty, higher levels of uncertainty may simultaneously decrease happiness, reflecting a human discomfort with ambiguity [48]. Fostering curiosity in a low-stakes environment, like art museums, where artwork allows for multiple interpretations and uncertainty is celebrated rather than feared, may help medical students find happiness in embracing ambiguity. For instance, positive humor and playful discussions with peers regarding interpretations of artwork, such as those of satirical cartoonists, may help learners cope with the uncertainties encountered in training [49,50]. As another example, encouraging students to engage with abstract or surrealist art can promote open-ended exploration, reignite the joy of curiosity and wonder, and encourage information-seeking rather than fear in the face of uncertainty—ultimately contributing to greater happiness during training [51].

Art museum-based education also facilitates self-reflection. A Johns Hopkins study on reflective practices in museum settings found that engaging with art can assist students in answering subjective, complex questions like “What does it mean to live a good life?” [52,53] for both themselves and their future patients. These guided reflections challenge students to connect their professional aspirations with broader existential questions, fostering clarity and life satisfaction as they progress through training and make critical career decisions.

## Meaning and Purpose; Character and Virtue

Viewing and discussing visual artwork can also support medical students in understanding the historical and social contexts of disease [36,54,55]. Works like Frida Kahlo’s *The Broken Column (1944)* powerfully depict her chronic pain after spinal surgery, presenting a haunting self-portrait of her vulnerability and emotional suffering, as well as a critique of the pervasive misogyny of her era, a time when women’s pain was often dismissed as hysteria and society prioritized objectification over genuine acknowledgment of women’s struggles [56,57]. Today, patients continue to express their disease journeys through art, offering students a window into the grief, pain, and triumphs associated with conditions from cancer to mental illness [58,59,60]. Discussing these works without the immediate intention to diagnose or treat enables students to appreciate how illness and healthcare affects not just the body but also the soul [53,61,62,63]. In an era where emotional exhaustion in trainees is high, the human connection to caregiving can feel diminished [64]. Art museum-based education offers a counterbalance, rekindling students’ emotional and relational purpose in pursuing a career of healing while developing coping skills to handle these heavy emotions [61,65].

Art museum-based learning activities can also help students explore themes of culture and inequality in an artist’s work, while openly challenging preconceived biases alongside peers. Many artworks engage with ethical tensions, portray the suffering and inequality of societies past and present, or depict moral conflicts. Programs like Yale’s Making the Invisible Visible encourage medical students to engage with artwork to examine the structures and hierarchies they will encounter in their careers [36]. By confronting themes of bias, racism, and power through works such as Kerry James Marshall’s *Untitled (2009)*, students are reminded of the broader responsibilities of inclusivity and ethics inherent in being a physician [36,66]. In learning to approach artwork with an unbiased and open mind to uncover its subtle nuances and differences from one’s own experiences, students can apply these same insights to building patient relationships as well. For some students, these artworks may also act as mirrors, resonating with the barriers they faced in navigating a career in medicine [36].

Museum-based activities not only combat the cynicism and instrumentalism that can arise from focusing solely on publishing papers, attaining high scores on examinations, and technical mastery, but can offer students a renewed sense of meaning and purpose, as well as an opportunity to cultivate character and virtue. These activities can help students connect with a meaningful desire to improve representation in medicine, combat injustice, and advocate for equitable care that understands the whole patient [67]. One powerful method for discussing these topics is through the “third thing:” an item–like a work of art–that exists outside of oneself and mediates reflection [68]. Third things can allow students to become aware of their own biases and inspire them to combat these biases in themselves and others and work towards dismantling systemic barriers in healthcare [19,68]. Students may even engage in their own visual art activism, as was done in a week-long elective for fourth-year medical students at McGovern Medical School [69]. By reconnecting students to their foundational motivations, such as alleviating suffering, fostering human connection, and advancing societal well-being, art museum-based education reframes medical training as a pathway more than just excelling in assessments.

## Close Social Relationships

Museum-based learning activities often involve group-based or paired activities in which students work together, with faculty guides, to engage in individual reflection and open-ended discussion prompted by artworks. These group conversations encourage the sharing of various perspectives. For example, one student may focus on an artwork’s use of color, another on its composition, and another on its symbolic elements, working together to form a cohesive narrative. In the context of a career in medicine, this mirrors the appreciation of multiple perspectives and multidisciplinary collaboration needed on healthcare teams. Studies have found that art museum-based programs can strengthen team building, foster cultural humility, and encourage active and reflective listening skills [70,71].

Participation in activities such as viewing art in galleries and museums has also been shown to strengthen interpersonal relationships and reduce feelings of loneliness [47,72,73,74]. For medical students who face high levels of burnout and emotional exhaustion, these experiences offer a valuable opportunity to build connections with both peers and faculty in a relaxed, non-clinical setting. These newly formed social networks can carry over into clinical spaces, fostering a sense of camaraderie and mutual support during rotations, or even encourage more robust connections with patients. Art museum-based activities can also nurture emotional strength through shared reflection. Working in pairs or small groups to interpret ambiguous works of art develops skills in respectful debate and builds a sense of community over time, promoting both individual and collective well-being.

## Challenges

Common arguments against the incorporation of the arts and humanities into medical education, including art museum-based activities, include the need to prioritize clinical content and skills in an already packed curriculum, weak integration with traditional curricula and a lack of standardization in such courses, uncertainty around optimal learner assessment, trouble in acquiring funding, and worries that students may not take “feelings” courses seriously [75,76]. Yet, studies of brief elective courses show that even small interventions can enhance the non-technical skills needed in medicine without detracting focus from biomedical and clinical courses [23,35,36].

Moreover, although the focus of this paper is on flourishing domains, many of these domains overlap with published medical learner frameworks such as the Master Adaptive Learner model, which highlights the importance of curiosity, meaning-making, and reflection in professional development for medical learners [77]. For example, the domain of Character and Virtue reflects the Master Adaptive Learner model’s emphasis on reflection and critical self-assessment. Close Social Relationships align with the interpersonal dimensions of learning emphasized in “Assessing” and “Adjusting” phases, which focus on formal and informal feedback, collaboration, and shared meaning-making. Lastly, Meaning and Purpose relates to the model’s “Planning” phase, which centers on identifying meaningful gaps in knowledge and being motivated by curiosity and purpose to grow. Therefore, art museum-based education in the context of fostering flourishing offers another innovative and complementary approach to support the development of clinically relevant skills and attitudes in medical learners.

Prior studies have found that medical students who participated in visual training using paintings performed significantly better in diagnoses and clinical skills than those students who did not [78,79]. VTS, a widely used approach in museum-based education, provides a structured framework for facilitating open-ended discussions prompted by a work of art, which ensures methodological consistency while allowing room for creativity [24]. Just as every patient presentation does not reflect the exact textbook description of a diagnosis, artworks rarely come with a single, correct interpretation. In VTS discussions, learners encounter multiple credible readings of the same piece and must communicate clearly together to consider these various interpretations and revise their ideas. Over time, such art museum-based discussions may lead to improved tolerance for ambiguity and empathy, leading to better patient satisfaction, adherence, and trust [80]. Conveying these benefits and optimizing the timing and perhaps non-elective nature of these museum-based activities in the medical curricula may help students take these programs more seriously, especially when they see the direct connections between the skills and attitudes developed through art museum-based education and their success as clinicians.

Integration of art museum-based activities may also pose a logistical barrier, such as when no museums are located near a medical school, or students do not have access to transportation, or when too few educators are trained to facilitate these activities. Where access to major museums is limited, classroom or virtual discussions that use digital images are acceptable options and may provide similar, although perhaps not identical, benefits to medical learners [81,82]. For example, virtual formats may limit opportunities for spontaneous peer interaction, reduce the sense of shared presence, and constrain viewers’ ability to observe artwork from multiple angles or experience its scale and texture. Nevertheless, when thoughtfully facilitated, these adaptations can still support core educational goals such as close observation, reflective dialogue, and perspective-taking. And faculty development programs focused on developing health professions educators’ skills in art museum-based teaching are increasingly available [83].

## Conclusion

As the demands of physician training intensify, the ability to flourish both personally and professionally is increasingly essential. Art museum-based medical education offers an engaging, distinctive, and evidence-informed approach to fostering this growth in medical students. By enhancing physical health, comfort with uncertainty, emotional intelligence, teamwork, and emotional well-being, these interventions align with VanderWeele’s model’s domains of flourishing. Integration of the arts and humanities generally, and museum-based learning specifically, into medical education has the potential to produce physicians who deeply understand the human condition and the significance of the profession of medicine. This approach cultivates physicians who are able to form meaningful connections with colleagues and patients to deliver exceptional patient care with integrity, empathy, and precision. To support the flourishing of their future patients, as well as to sustain their own well-being as physicians, medical learners must look beyond the study of disease processes and engage with the complex personal and social contexts that shape human flourishing.
